# Feeding a High Concentrate Diet Down-Regulates Expression of ACACA, LPL and SCD and Modifies Milk Composition in Lactating Goats

**DOI:** 10.1371/journal.pone.0130525

**Published:** 2015-06-18

**Authors:** Hui Tao, Guangjun Chang, Tianle Xu, Huajian Zhao, Kai Zhang, Xiangzhen Shen

**Affiliations:** College of Veterinary Medicine, Nanjing Agricultural University, Nanjing, 210095, China; University of Lleida, SPAIN

## Abstract

High concentrate diets are fed to early and mid-lactation stages dairy ruminants to meet the energy demands for high milk production in modern milk industry. The present study evaluated the effects of a high concentrate diet on milk fat and milk composition, especially, *cis*-9, *trans*-11 CLA content in milk and gene expression of lactating goats. Eight mid-lactating goats with rumen fistula were randomly assigned into a high concentrate diet (HCD) group and low concentrate diet (LCD) group. High concentrate diet feeding significantly increased lipopolysaccharides (LPS) in plasma and decreased milk fat content, vaccenic acid (VA) and *cis-9*, *trans-11 *CLA in milk of the lactating goats. The mRNA expression levels of sterol regulatory element binding protein B 1c (SREBP1c), lipoprotein lipase (LPL), fatty acid synthetase (FASN) and acetyl-CoA carboxylase α (ACACA, ACCα) involving in lipid metabolism were analyzed, and ACACA and LPL all decreased in their expression level in the mammary glands of goats fed a high concentrate diet. DNA methylation rate of stearoyl-CoA desaturase (SCD) was elevated and decreased, and SCD mRNA and protein expression was reduced significantly in the mammary glands of goats fed a high concentrate diet. In conclusion, feeding a high concentrate diet to lactating goats decreases milk fat and reduced expression of SCD in the mammary gland, which finally induced *cis-9*, *trans-11* CLA content in milk.

## Introduction

Diet plays an important role in the modulation of fatty acid composition in ruminant products. The types and proportion of fatty acids in food have different effects on human health maintenance and disease prevention [1]. Polyunsaturated fatty acids (PUFAs) are fatty acids containing two or more double bonds. In particular n-3 fatty acids, which first double bond is 3 carbons from the methyl end of the fatty acid chain, includes linolenic acid (LNA, C18:3n-3), eicosapentaenoic acid (EPA, C20:5n-3), docosapentaenoic acid (DPA, C22:5n-3) and docosahexaenoic acid (DHA, C22:6n-3) and so on, and the main sources of n-3 PUFA are fish [2]. n-6 PUFA, of which first double bond is 6 carbons from the methyl end of the fatty acid chain, includes linoleic acid (LA, C18:2n-6), γ-linolenic acid (GLA, C18:3n-6), dihomo-γ-linolenic acid (DGLA, C20:3n-6), arachidonic acid (AA, C20:4n-6) and so on, and n-6 PUFA arevegetable oils [3]. It is recommended intake of n-6/n-3 PUFAs for human diets is 2.3:1, but in many countries, diets deficient in n-3 fatty acids and too many of n-6 fatty acids results in the ration ranging from 15:1 to 20:1 [4]. Besides that, consumers are becoming more aware of the optimal fatty acid profile of ruminant food and not just the simple fat content. Conjugated linoleic acids (CLA) are a mixture of positional and geometric isomers of octadecadienoic acid with conjugated double bonds [5]. CLA isomers have potential benefits for human health, such as the inhibition of cancer and fat deposition. *Cis-9*, *trans-11* CLA, known as rumenic acid (RA), is the predominant isomer that comprises up to 90% of total CLA [6]. There are two sources of *cis-9*, *trans-11* CLA in ruminants. One source is ruminal biohydrogenation of linoleic aid to stearic acid in the rumen [7,8], and the other source is the endogenous conversion of *tran-11* C18:1 (vaccenic acid, VA) in mammary glands and ruminant adipose tissue [9,10].

Many studies focused on an increase in *cis-9*, *trans-11* CLA content in ruminant products by feeding different dietary substrates. The feeding of linoleic acid- and linolenic acid-enriched diets increased essential fatty acids in beef [11]. Fish oil increased the duodenal flow of long-chain polyunsaturated fatty acids and *trans-11* C18:1 and decreased C18:0 in steers via changes in the rumen bacterial community [12]. Stearoyl coenzyme-A desaturase (SCD) is the primary enzyme involved in *cis9*, *trans11*-CLA endogenous synthesis, which introduces a double bond in the Δ9 position of the precursor, *trans-11* vaccenic acid (*trans-11* C18:1, VA) [13]. The activity of this enzyme in the mammary gland produces 90% of the CLA formed in milk [14]. Dairy cows or goats are usually fed a high concentrate diet during early and mid-lactation stages to meet the energy demands for high milk production [15]. This feeding practice might improve high milk yield or rapid weight gain in short term. However, the over feeding of dairy cows or dairy goats with a high concentrate diet results in severe metabolic disorder, which is defined by SARA(Subacute Ruminal Acidosis) as pH value of ruminal fluid below 5.6 for at least 3 hours per day or 5.8 [16,17]. Numerous Gram-negative bacteria disrupt and release large amounts of endogenous lipopolysaccharides (LPS) under SARA status [18]. Free LPS could translocate into the bloodstream and initiate an inflammatory response, which reduces productivity of the ruminants [19]. Intensive production systems in ruminants tend to include a high proportion of grains and digestible carbohydrates in the diet. Recent studies showed that induced SARA altered the milk fatty acid profile in dairy cows The milk fat percentage was decreased but milk protein increased in ruminants suffered from SARA, also concentration of C18:0 and c9t11CLA decreased, and so on. [20, 21] In a recent study, we indicated that long-term SARA affected milk fatty acid composition [22]. However, less information is available on *cis-9*, *trans-11* CLA synthesis in ruminants suffering from a high concentrate diet-induced SARA. Therefore, the present study evaluated the effects of *cis*-9, *trans*-11 CLA content in the milk of lactating goats fed a high concentrate diet, and the changes of other milk composition and relative genes in mammary gland. Some key enzymes also influence lipid metabolism. Therefore mRNA expression levels of sterol regulatory element binding protein B (SREBP), lipoprotein lipase (LPL), fatty acid synthetase (FASN), acetyl-CoA carboxylase α (ACACA) and stearoyl-CoA desaturase(SCD) were analyzed in this study.

## Materials and Methods

### Ethics statement

The Animal Care and Use Committee of Nanjing Agricultural University approved the experimental protocol, which was performed in accordance with the Guidelines for Experimental Animals of the Ministry of Science and Technology (2006, Beijing, China).

### Animals and experimental design

Eight healthy multiparous mid-lactating goats (average body weight, 43.8±2.6 kg) with rumen fistula were randomly assigned to two groups. Goats were housed in pens and fed total mixed rations that were formulated to meet nutritional requirements. Goats in control group were fed a low concentrate diet (LCD) with a forage-to-concentrate ratio (F:C) of 6:4. Goats in the HCD group were fed with a high concentrate diet (HCD) with an F:C of 4:6. Diet formulas are shown in Table 1. 0.4kg dry matter (DM) were offered to goats twice a day at 07:00 and 19:00, and goats were free access to fresh water. Dry matter intake (DMI) was measured. The feeding adaption period was 3 weeks. Goats were milked at 07:00 and 19:00 h daily, and milk yield was recorded.

**Table 1:** Ingredients and nutrient composition of the diets.

Items	The ratio of forage to concentrate	
	6: 4	4: 6
Ingredient (% of DM)		
Chinese wild-rye hay	48.00	32.00
Alfalfa hay	12.00	8.00
Corn	29.20	23.07
Wheat bran	0.00	28.30
Soybean meal	8.43	2.00
Rape seed meal	0.00	3.70
Limestone meal	0.57	1.43
Calcium phosphate dibasic	0.90	0.60
Salt	0.40	0.40
Premix*	0.50	0.50
Total	100	100
Nutrient level (% of DM)		
Net energy (MJ/kg)	17.17	17.56
Crude protein (%)	17.79	16.76
Fat (%)	2.39	3.60
Neutral detergent fiber (%)	40.97	42.64
Acid detergent fiber (%)	2.83	7.22
Calcium (%)	1.73	1.55
Phosphorus (%)	0.79	0.76

*Premix ingredients (per kg diet), V_A_:6000U, V_D_:2500U, V_E_:80mg, Cu:6.25mg, Fe:62.5mg, Zn:62.5mg, Mn:50mg, I:0.125mg, Co:0.125mg, Mo:0.125mg.

### Sample collection

Days were counted from day 1 to day 24 as the goats were adapted with the diets. Milk samples were taken from morning milking on day 22. One aliquot was stored at 4°C until it was analyzed for milk components, and a second aliquot was stored at -70°C until analyzed for fatty acid composition. Blood samples were collected 2 h after the morning feeding via jugular veins using vacuum tubes containing sodium heparin as anticoagulant on day 23. Blood samples were centrifuged at 1900 g, 4°C for 15 min, and plasma was harvested. Ruminal fluid samples were taken 2 h after morning feeding and strained through a double-layered muslin gauze on day 24 for fatty acids analysis. Mammary gland tissues were obtained by biopsy after 2 h after the morning feeding on day 25 of the experimental period. Local anesthesia (lidocaine hydrochloride) was administered into breast skin in a circular pattern surrounding the incision site, then 2cm incision was made and mammary gland tissue was dissected. Tissue samples (500–1000 mg) were rinsed with 0.9% saline, snap frozen in liquid nitrogen and subsequently stored at −70°C until RNA, DNA and protein extraction. Incisions were sutured, and antibiotics were administered intramuscularly to avoid infection. After slaughtered, *longissimus* muscle, Semitendinosus muscle and subcutaneous fat were collected for extraction of total lipids.

### Measurement of rumen, plasma and milk parameters

Rumen pH was measured using a pH meter on day 22, and the duration of pH value lower than 5.6 and 6.0 was recorded. LPS content in rumen fluid and plasma was determined using a chromogenic endpoint assay (Chinese Horseshoe Crab Reagent Manufactory Co., Ltd., Xiamen, China) with a minimum detection limit of 0.01 EU/mL. These procedures were performed according to the manufacturer’s instructions. Milk samples were taken to determine milk components (MilkoScan FT1, FOSS, Denmark) and somatic cell count (SCC) (Fossomatic 5000, Denmark).

### Fatty acids composition analysis

Volatile fatty acids anylysis. Acetate, propionate, butyrate, isobutyrate, and isovalerate concentrations were determined by using gas chromatography (Agilent Technologies 7890A, GC system) with a flame ionization detector, based on the method with some modifications [23]. Crotonic acid was used as internal standard, and chloroform was served as extracting solvent. And the gas chromatography was performed using a 30 m×0.320 mm×0.50 μm fused silica capillary column (J&W Science 123–3233, USA). Helium was used as the carrier gas and hydrogen as a fuel gas at the flow rate of 40 mL/min with air as a combustion-supporting gas. The temperature of the column, the flame ionization detector and the injector were 155°C, 180°C and 180°C, respectively. The split ratio was 20:1 and 1 μL was injected. Crotonic acid was served as internal standard, and chloroform were used to extract VFA in ruminal fluid.

Long-chain fatty acids analysis. Total lipids of ruminal fluid, plasma, muscle, subcutaneous fat and milk were extracted from samples using a mixture of polar and non-polar solvents according to Folch *et al*. [24]. Given no significance in total lipids of muscle and subcutaneous fat between two groups, fatty acids composition of them was not further detected. Fatty acid methyl esters (FAME) were prepared by esterification using sodium methoxid, followed by 14% borontrifluoride in methanol [25]. Heptadecanoic acid methyl ester served as an internal standard, and it was added to samples prior to extraction and methylation.

FAME extracts were used for gas chromatographic analysis of total fatty acids. Fatty acid composition was determined using gas chromatography (GC) on a CP SIL 88, 100 m×0.25 mm×0.25 μm capillary column (Agilent J&W Advanced Capillary GC Columns, Netherlands) in an Agilent 7890A (Agilent Technologies, USA) with an auto sampler, flame ionization detector and split injection. The initial oven temperature was 150°C, which was held for 5 min then increased to 200°C at a rate of 2°C/min and held for 10 min, then increased to 220°C at 5°C/min and held for 35 min. Helium was used as a carrier gas at a flow rate of 1 mL/min. The injector was set at 260°C, and the detector was set at 280°C. FAMEs were identified by comparisons with the retention times of the standards. The standards were FAME Mix C4-C24 Unsatures (Sigma 18919, Germany), Methyl *trans*-11 C18:1 (Sigma 46905, Germany) and Methyl *cis*-9, *trans*-11 CLA (Matreya1255, USA).

### RNA extraction and analysis

Total RNA was extracted from 50 mg mammary gland tissues in RNAiso Plus reagent (Takara Co., Otsu, Japan) via homogenization on ice. The purity and concentration of RNA were measured using Eppendorf BioPhotometer Plus (Eppendorf AG, Hamburg, Germany). First-strand cDNA of each sample was synthesized using 500 ng of total RNA template in PrimeScript RT Master Mix Perfect Real Time (Takara Co., Otsu, Japan). All templates were performed in triplicate, and glyceraldehyde phosphate dehydrogenase (GAPDH) and β-actin served as internal control genes for normalization. Primers were designed using Primier 6.0 (Premier Biosoft International, USA) or online tool PrimerQuest of Integrated DNA technologies Co. Ltd (http://www.idtdna.com/Scitools/Applications/Primerquest/) based on known goat sequences (shown in Table 2). qPCR was performed using SYBR Premix Ex Taq (Takara Co., Otsu, Japan) and an ABI 7300 Real-Time PCR System (Applied Biosystems, Foster City, CA, USA) according to the instruction manual’s recommendations. Each sample was measured in triplicate. Data were analyzed using the 2^-ΔΔCt^ method using the geometric means of the selected housekeeping genes, GAPDH and β-actin, for normalization according to the strategy described previously [26].

**Table 2:** The primer sequences of the target and the internal control genes in *Capra Hircus* used in qPCRs.

Gene name	Gene bank accession	Sense primer	Antisense primer
ACACA	DQ370054.1	TGCAGGAGGGCTTCATGAATTTGC	TCAACTGGAAGCTTTCCGTCTCCA
SCD	AF422171.1	ACAAAGGTTCCCAGAGAGCCATGA	TCCCTAGCAGAATGCCTCACACTT
FASN	DQ915966.3	CATCCTCGCTCTCCTTCA	CGCCTGTCATCATCTGTC
LPL	DQ370053.1	ACGAGCGTTCCGTTCATCTCTTCA	TAGCCCATGTTGTTGCAACGGTTC
SREBP1	JN790254.1	TGGGCACTGAGGCCAAGTTGAATA	ACTCAGGTTCTCCTGCTTGAGCTT
β-actin	AF481159.1	ACCACTGGCATTGTCATGGACTCT	TCTTCATGAGGTAGTCCGTCAGGT
GAPDH	AJ431207.1	CATGTTTGTGATGGGCGTGAACCA	TGATGGCGTGGACAGTGGTCATAA

### DNA methylation assay of SCD

Total DNA from mammary gland tissues was extracted using a commercial kit (Invitrogen, California, USA), and sodium bisulfate and Hydroquinone solutions were added to the DNA for getting bisulfate-converted DNA according to Ogino’s method [27]. DNA methylation was assayed using bisulfite sequencing PCR (BSP) (BBI, Canada). DNA was treated with bisulfate to convert unmethylated cytosines(C) to uracils(U) through deamination, but methylated cytosines remained unchanged during treatment. Uracils were eventually replaced by thymine (T) during PCR amplification. The modified target sequences were amplified by PCR with primers, 5'-GTAGYGGAAGGTTTYGAGTATAG-3' (forward) and 5'-TTCCCAACAAACTAAAAACAATAC-3' (reverse) that specifically bind to the plus strand of the corresponding genomin regions. This causes the occurrence of C/T polymorphisms at these positions in subsequent sequencing reactions. PCR amplification made a mixture of molecules with either C or T bases at CpG (cytosine-phosphate-guanine) dinucleotides. PCR products were cloned and sequenced to determine DNA methylation levels of the SCD gene using BiQ Analyzer software by Shanghai Sangon Biotech Company (Shanghai, China).

### Western blotting analysis

Protein was extracted after homogenizing mammary gland tissue, and the concentrate was assayed using the Bradford method [28]. Proteins were separated using SDS-PAGE and transferred to a nitrocellulose membrane (Millipore, USA). The membrane that included proteins <50 kDa was incubated with a specific primary antibody, polyclonal antibody raised in goat (sc-23016, Santa Cruz, USA), and the membrane that included proteins >50 kDa was normalized against β-tubulin and incubated with a polyclonal goat antibody (sc-9935, Santa Cruz, USA). Membranes were incubated overnight at 4°C and re-probed with a horseradish peroxidase-conjugated Affinipure rabbit anti-goat secondary antibody (E030130-01, Earthox LLC, San Francisco, CA). Protein bands were visualized using a chemiluminescent agent (BeyoECL plus, Shanghai, China). Membranes were scanned using a chemiluminescence imager, Image Quant LAS 4000 (GE, USA), and band intensities were densitometrically evaluated using Quantity One software (Bio-Rad, USA). Signal intensities of the samples are expressed as a percentage of the reference sample.

### Statistical Analysis

The data were analyzed using general linear model (GLM) with random goat effect and the fixed factor of diets (SAS Systems, SAS Institute Inc., Cary, NC), and adjusted means were compared with a Student’s t-test. Data were presented as least squares mean (LSM) and the standard error (SE), and all statistical tests were performed for a significance level *P*<0.05.

## Results

### Rumen, plasma and milk parameters

Different diets had no influence on DMI of goats, milk yield, total lipids in muscle and subcutaneous fat (Table 3). The pH value of ruminal fluid in the HCD group was significantly lower than in the LCD group (*P*<0.05). The pH was less than 5.6 for more than 3 h one day (212±29.15 min, shown in Table 3), which indicated that the high concentrate diet induced SARA status in goats in the HCD group. Milk component assays showed that milk fat in the HCD group was significantly declined (*P*<0.05). Somatic cell count tended to increase in the HCD group, but this increase was not significant. LPS content in ruminal fluid tended to be elevated in the HCD group, but, it was significantly increased in plasma (*P*<0.05) (Table 3).

**Table 3:** Rumen, plasma and milk parameters.

Item	LCD group	HCD group	*P*-value
Rumen pH			
Average pH	6.25±0.08	6.03±0.02	*
Minimum pH	5.82±0.03	5.54±0.06	*
Time<pH5.6, min/d	0.00	212±29.15	**
Time<pH6.0, min/d	128±31.56	330±28.17	-
Milk parameter			
Yield(kg/d)	0.68±0.052	0.67±0.0637	-
Fat (%)	3.90±0.11	2.95±0.13	**
Protein (%)	3.06±0.10	2.97±0.12	-
Lactose (%)	4.74±0.09	4.89±0.06	*
Total solids content (%)	12.60±0.15	12.01±0.09	**
Nonfat milk solids (%)	8.40±0.07	8.57±0.07	-
Urea (mg/dL)	21.29±0.69	21.32±0.76	-
Somatic cell count (10^3^/mL)	2710.13±865.67	3583.91±1167.32	-
LPS content, EU/mL			
Ruminal fluid	38854±2175	48064±3213	-
Plasma	0.89±0.03	1.07±0.04	**
Fat(%) in muscle and Subcutaneous fat tissue			
Longissimus muscle(mg/g)	2.82±0.143	2.90±0.206	-
Intramuscular muscle(mg/g)	0.92±0.071	1.06±0.053	-
Subcutaneous fat(mg/g)	49.53±2.392	48.76±3.157	-

- means non-significant

* *P*<0.05

** *P*<0.01

### Fatty acid composition analysis

VFA compositions of ruminal fluid are shown in Table 4. Acetate concentration in HCD group was lower than that in control group (*P*<0.05), and propionate higher than that in control group (*P*<0.05)

Total long chain fatty acid compositions of ruminal fluid, plasma and milk are shown in Table 5. Diet had no effect on VA content in ruminal fluid, but *cis-9*, *trans-11* CLA content decreased significantly in the HCD group compared to the control group (*P*<0.01). The contents of C18:0 (*P*<0.05) and C20:1 (*P*<0.05) in ruminal fluid were higher in the HCD group than the control group. The content of C18:3n3 (*P*<0.05), C22:0 (*P*<0.01) and C24:1 (*P*<0.01) in ruminal fluid was lower in the HCD group than the control group.

**Table 4:** VFA composition in ruminal fluid.

Item	LCD group	HCD group	*P*-value
Acetate(C2:0) mmol/L	28.83±1.494	22.38±1.652	*
Propionate(C3:0) mmol/L	18.42±1.906	23.61±1.593	*
Butyrate(C4:0)) mmol/L	9.51±1.101	8.73±0.657	-
Total VFA mmol/L	64.05±5.046	62.85±3.285	-
A/P	1.59±0.091	1.08±0.060	*

- means non-significant

* *P*<0.05

** *P*<0.01

**Table 5:** Fatty acid composition in the ruminal fluid, plasma and milk.

Item	Fatty acids in rumimal fluid (mg/g total lipids)			Fatty acids in plasma (mg/g total lipids)			Fatty acids in milk (mg/g total lipids)		
	LCD group	HCD group	*P*-value	LCD group	HCD group	*P*-value	LCD group	HCD group	*P*-value
C12:0	0.35±0.03	0.37±0.06	-	0.54±0.00	0.51±0.04	-	8.13±0.61	9.76±0.72	-
C14:0	2.15±0.36	2.27±0.35	-	7.85±0.37	7.69±0.25	-	14.12±1.37	17.46±1.44	-
C14:1	Not Det.			1.56±0.03	1.62±0.04	-	0.79±0.06	0.75±0.04	-
C16:0	40.92±1.96	39.36±2.03	-	89.36±9.72	78.64±5.61	*	39.50±3.49	41.67±2.64	-
C16:1	Not Det.			5.12±0.64	6.05±0.35	*	1.73±0.25	2.01±0.27	-
C18:0	78.36±6.35	90.65±8.62	*	103.43±10.26	91.38±10.30	*	10.57±0.36	9.30±0.98	*
t9C18:1	0.86±0.06	0.97±0.11	-	4.26±0.54	3.70±0.16	**	0.42±0.02	0.21±0.02	*
t11C18:1(VA)	5.18±0.76	5.36±0.61	-	5.91±0.99	3.79±0.63	**	1.32±0.11	0.60±0.09	**
C18:1n9c	8.94±1.59	8.46±1.14	-	159.63±10.37	162.35±9.18	-	27.39±1.78	25.37±1.60	-
C18:2n6t	0.33±0.02	0.30±0.03	-	2.09±0.06	1.46±0.36	**	0.02±0.00	0.02±0.00	-
C18:2n6c	10.07±2.24	11.76±2.91	-	169.85±10.63	183.86±17.31	**	3.67±0.19	3.02±0.16	*
C20:0	0.71±0.07	0.74±0.10	-	Not Det.			0.34±0.03	0.21±0.03	*
C18:3n6	Not det.			1.58±0.35	1.89±0.43	**	0.04±0.00	0.05±0.01	*
C20:1	0.21±0.01	0.30±0.01	**	1.20±0.23	1.20±0.33	-	0.08±0.00	0.08±0.01	-
C18:3n3	1.23±0.09	1.06±0.07	*	10.33±0.64	6.75±0.95	**	0.69±0.05	0.44±0.03	**
C9t11CLA	0.98±0.30	0.60±0.26	**	2.16±0.17	1.23±0.09	**	1.10±0.10	0.70±0.05	**
C20:2	Not Det.			0.98±0.08	0.95±0.12	-	0.03±0.00	0.03±0.00	-
C20:3n6	Not Det.			1.36±0.15	1.37±0.18	-	0.05±0.01	0.04±0.00	-
C22:0	0.90±0.07	1.26±0.07	**	Not Det.			0.09±0.01	0.06±0.01	**
C20:3n3	Not Det.			50.52±2.47	38.14±1.96	**	0.69±0.02	0.53±0.04	*
C20:4n6	Not Det.			Not Det.			0.02±0.00	0.04±0.01	*
C22:2	Not Det.			Not Det.			0.04±0.00	0.05±0.00	*
C20:5n3	Not Det.			8.33±0.46	6.13±0.71	**	0.10±0.01	0.07±0.01	**
C24:1	1.53±0.10	1.24±0.09	**	2.86±0.23	2.87±0.16	-	0.08±0.08	0.09±0.01	-
C22:6n3	Not Det.			9.38±0.87	7.53±0.26	**	0.38±0.03	0.18±0.02	**
SFA	123.61±7.53	134.93±9.76	-	202.86±10.28	178.49±12.39	*	73.78±4.57	78.50±3.55	-
MUFA	16.81±2.92	16.42±1.98	-	180.52±15.23	181.58±13.71	-	31.83±4.26	29.19±2.90	-
PUFA	12.76±3.19	13.62±3.51	-	266.88±11.62	249.34±20.36	-	6.46±0.06	5.21±0.09	-
n-6/n-3	8.53±0.15	11.38±1.80	**	2.23±0.05	3.22±0.07	*	2.04±0.0.16	2.60±0.08	-

- means non-significant, “Not Det.” means no material was detected

* *P*<0.05

** *P*<0.01

The content of VA (*P*<0.01) and *cis-9*, *trans-11* CLA (*P*<0.01) in plasma was remarkably decreased in the HCD group than the control group. The contents of C16:0 (*P*<0.05), C18:0 (*P*<0.05), *trans-9* C18:1 (*P*<0.01), C18:2n6t (*P*<0.01), C18:3n3 (*P*<0.01), C20:3n3 (*P*<0.01), C20:5n3 (*P*<0.01) and C22:6n3 (*P*<0.01) in plasma were reduced in the HCD group compared to the control group. However, the levels of C16:1 (*P*<0.05), C18:2n6c (*P*<0.01), C18:3n6t (*P*<0.01) were obviously increased in the HCD group. n-6/n-3 ratio in milk of HCD group had no significant difference between the two groups. The diet had no effect on MUFA and PUFA in milk, but decreased and the ratio of n-6 to n-3 PUFA (*P*<0.05) in milk. The content of VA (*P*<0.01) and *cis-9*, *trans-11* CLA (*P*<0.01) in milk samples was notably decreased in the HCD group compared to the control group. The following components, C18:0 (*P*<0.05), trans-9 C18:1 (*P*<0.05), C18:2n:6c (*P*<0.05), C20:0 (*P*<0.05), C18:3n3 (*P*<0.01), C22:0 (*P*<0.01), C20:3n3 (*P*<0.05), C20:5n3 (*P*<0.01) and C22:6n3 (*P*<0.01), were significantly declined in the HCD group compared to the control group. In contrast, the levels of C18:3n6 (*P*<0.05), C20:4n6 (*P*<0.05) and C22:2 (*P*<0.05) were enhanced in the HCD group.

### RNA extraction and analysis

**Fig 1:**
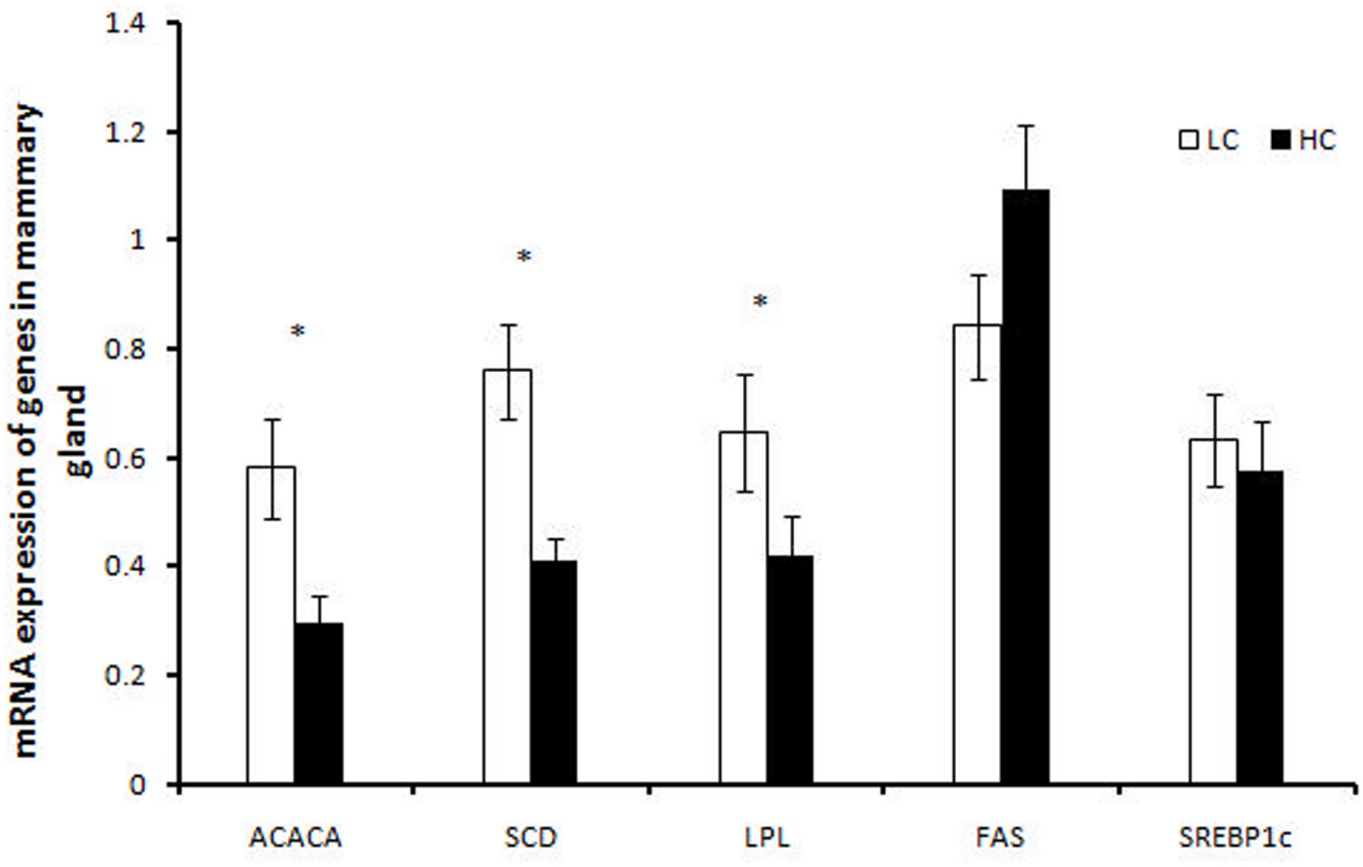
mRNA expression of genes involved in lipogenesis in mammary glands of lactating goats fed a high concentrate diet. The asterisk shows significant differences between the groups (*P*<0.05).

The effects of feeding different concentrate diets on mRNA expression levels of ACACA, SCD, LPL, FASN and SREBP1c are shown in Fig 1. The high concentrate diet in the HCD group significantly down-regulated transcription levels of ACACA, SCD and LPL mRNA compared to the control group (*P*<0.05). The expression of transcription factor SREBP1c tended to decrease in the HCD group, but this difference was not significant. FASN expression was not significantly different between the two groups.

### DNA methylation analysis of SCD gene

**Fig 2:**
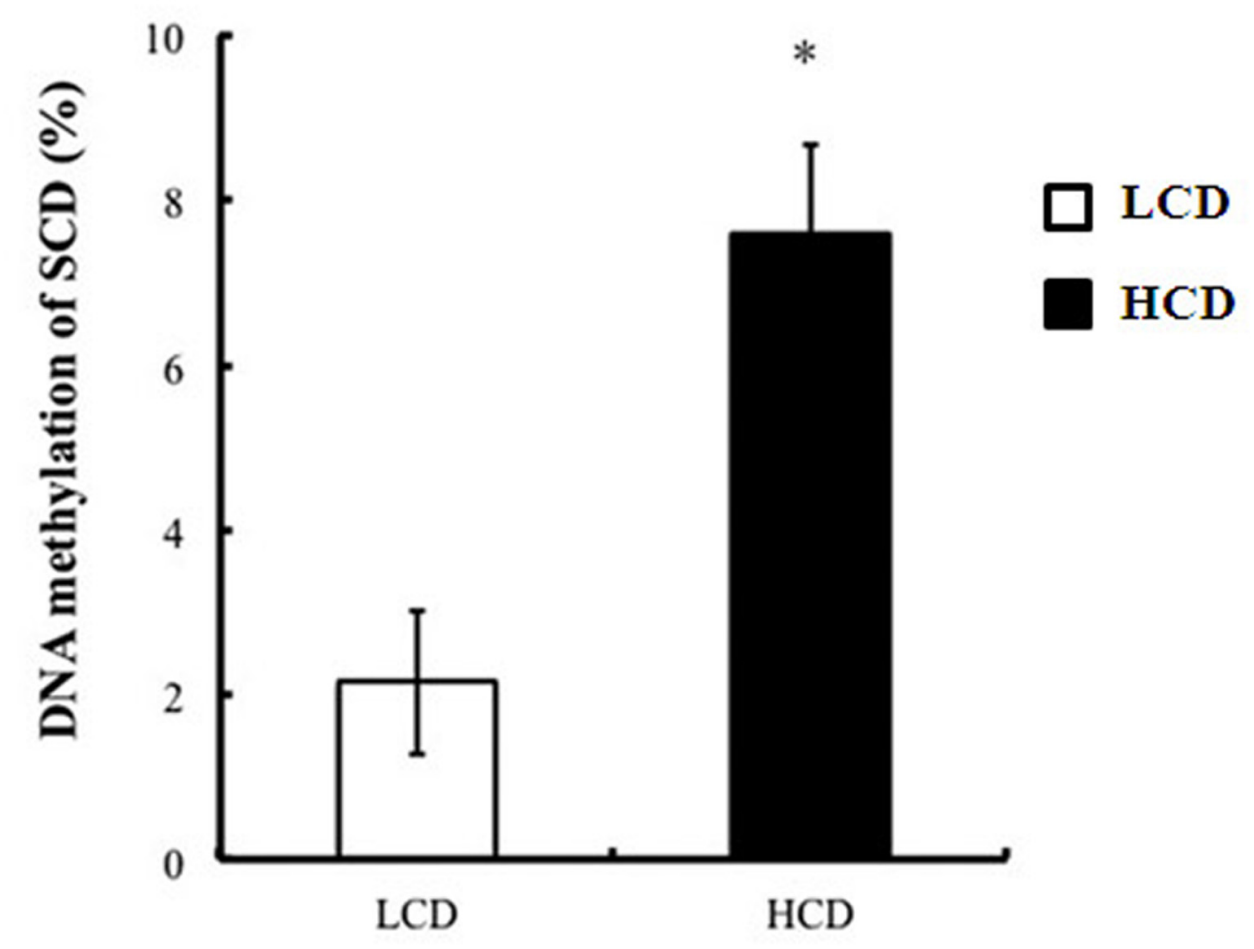
Frequency of DNA methylation on the CpG island in the promoter of SCD in the mammary glands of lactating goats fed a high concentrate diet. DNA methylation was analyzed using bisulfite sequencing PCR. Data represent the means and standard error, and the asterisk indicates statistical differences (*P*<0.05) between low and high concentrate diets.

DNA methylation of the SCD gene, which encodes a stearoyl-CoA desaturase in mammary glands that is involved in *cis*-9, *trans*-11CLA synthesis, was significantly increased in goats fed a concentrate diet compared to a low concentrate diet (Fig 2).

### Protein expression of SCD in mammary glands

**Fig 3:**
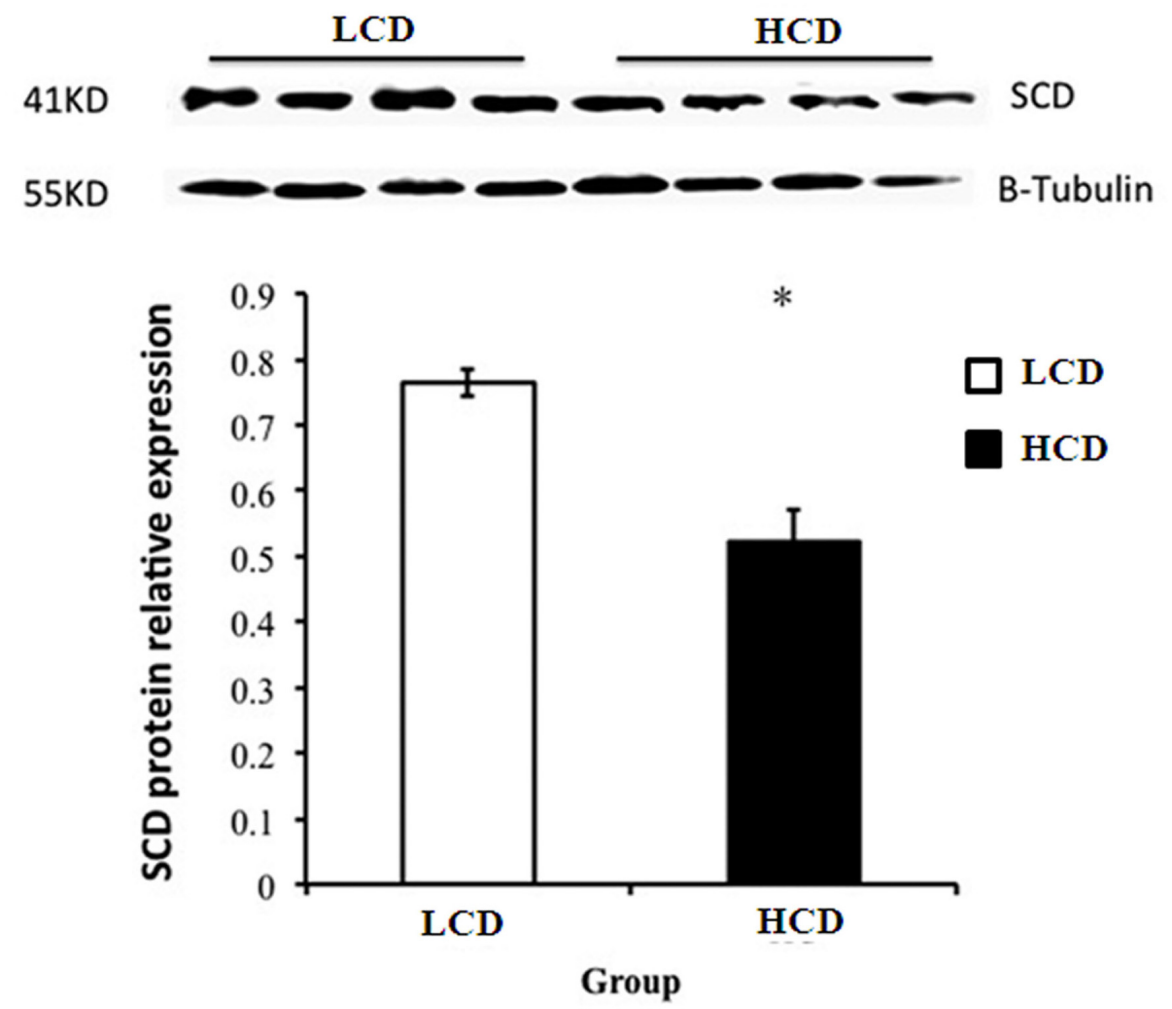
Protein expression of SCD in the mammary glands of lactating goats. Representative blots of SCD in mammary glands of goats fed a low concentration diet (lanes 1, 2, 3, 4) and a high concentration diet (lanes 5, 6, 7, 8). The asterisk shows significant difference between the groups (*P*<0.05).

Representative blots of SCD protein are shown in Fig 3. Expression levels of SCD protein in goat mammary glands was remarkably decreased in the HCD group compared to the control group fed a low concentrate diet. There was a significant difference between these two groups (*P*<0.05).

## Discussion

In the present study, high concentrate diet induced SARA status, as demonstrated by the pH value of ruminal fluid, which remained lower than 5.6 for more than 3 h in the HCD group.

Volatile fatty acids account for 60–70% metabolizeble energy supply in ruminants [29], and of the three main VFA, acetate and butyrate are essential components for synthesis of milk fat, while propionate, the only glucogenic VFA in lactating ruminants, is substrate for lactose synthesis [30]. In our study, acetate concentration in HCD group was lower than that in control group (*P*<0.05), and propionate higher than control group (*P*<0.05), which could finally influence the fat (*P*<0.01) and lactose (*P*<0.05) in milk. However, it was found that butyrate had greater influenced on milk yield than acetate and there was a positive regression relationship between A/P and milk fat [31], and in our study, the butyrate and milk yield were no significant between two groups. Goats suffered from SARA with low rumianl pH value may caused by high concentration of lactate, which was proved by our recent study in cows [32].

The supply of PUFA available for absorption is determined by both the amounts of PUFA in the diet and their metabolism in the rumen. In present study, the high concentrate diet had no effect on PUFA concentration in the ruminal fluid, which could result in no significant difference between the two groups in the milk PUFA. In grazing cow, increases the proportion of concentrate in diet from 30–350 g/kg dry matter resulted an increase C18:2n6 concentration but a reduction in C18:1n9c, c9t11CLA and C18:3n3 concentration in milk [33], and in the present study, it was found that high concentrate diet increased C18:2n-6 concentration (*P*<0.05) and reduced VA, c9t11 CLA and C18:3n3 concentration(*P*<0.05), and also reduced C18:1n9c, but without significance.

Previous studies showed that the quantity of biohydrogenation intermediates that are produced in the rumen affected their concentrations in milk [34]. VA is the precursor of *cis-9*, *trans-11* CLA synthesis in the mammary glands of ruminants [35]. Changes in the *cis-9*, *trans-11* CLA content in ruminant milk depend both on the processes involved in the biohydrogenation of diet-derived unsaturated fatty acids and on the availability of VA in the rumen. *Cis-9*, *trans-11* CLA is a transient intermediate in the rumen, whereas VA is an intermediate that accumulates when certain diets are consumed, which results in a lower conversion of *trans* C18:1 to C18:0. Goats in the HCD group suffering from SARA produced less *cis-9*, *trans11* CLA than goats in control group because the biohydrogenation of VA might be affected by the low pH value of ruminal fluid [36, 37, 38]. However, diet does not affect the concentration of *cis-9*, *trans-11* CLA in the rumen [39].

Plasma is the transportation system of nutritional ingredients, and its fatty acids status reflects the overall long-term lipid metabolism that is influenced by diet and rumen microbial activity. The profile of fatty acids in plasma represents the profile of fatty acids that are available to the mammary gland. The VA content in the plasma was significantly lower in the HCD group in present experiment, which might be caused by less absorption and more tissue utilization. Feeding pasture-based diets to German Holstein and German Simmental bulls’ results in significantly higher VA concentrations in plasma compared to concentrate-fed bulls [39]. High concentrate diet feeding in our study reduced VA concentration in plasma, and *cis-9*, *trans-11* CLA concentration in plasma was also lower in the HCD group, which might be due to lower absorption from the rumen.

The content of *cis-9*, *trans-11* CLA in milk was significantly lower in the HCD group, which might have resulted from less precursor and lower SCD expression in the mammary gland. The methylation rate of the SCD DNA promoter in the HCD group was up-regulated, which could down-regulated the expression of SCD, as a matter of fact the expression of SCD protein was significantly reduced in the HCD group than the control group, which indicated that less VA was desaturated into *cis-9*, *trans-11* CLA in the HCD group. The feeding of lactating goats with a high concentrate diet led to a low expression of SCD protein in mammary glands. These results suggest that the low expression of SCD protein in mammary glands leads to a low content of *cis-9*, *trans-11* CLA.

The administration of a high concentrate diet might improve high milk yield or rapid weight gain in the short term. However, long-term consumption of high concentrate diet is associated with disorders, such as SARA. This study demonstrated that a high concentrate diet decreased milk fat content and some milk fatty acid composition, which were good for human health.

LPL is an enzyme that hydrolyzes triacylglycerol (TAGs) to form fatty acids and glycerol. Long-chain fatty acids are taken up by mammary glands through the action of LPL, which markedly increases throughout lactation [40]. LPL hydrolyzes lipoprotein-associated triglycerides prior to fatty acid absorption by tissues, which could provide more fatty acids during milk fat depression [41]. In present study, the LPL mRNA expression was lower in mammary gland of goats fed on HCD, which indicated that less fatty acids were provided for milk fat synthesis in HCD condition, even more fat was in the diet. Our previous study demonstrated that the fat content in the milk was lower with a high concentrate diet, which is consistent with the abundant of LPL mRNA expression in LCD.

FASN, a complex multifunctional enzyme, plays an important role in energy homeostasis by converting excessively consumed food into lipids for storage and energy stimulation in β-oxidation [42]. FASN is also required for the production of milk lipids during lactation [43]. Palmitate (C16:0) is the major saturated constituent of lipids in animal tissues, and it is partially synthesized *de novo* by FASN [44]. C16:0 was lower in plasma in HCD group than that in control group (*P*<0.05), but had no difference in milk in two groups, which was consistent with the expression of FASN in mammary gland, expression of FASN was higher in HCD group, but without significance. FASN regulates lipid storage when high energy is consumed [42]. The HCD contained more fat than the low concentrate diet, which could affect FASN expression in the mammary gland of goats in HCD group. A previous study in lactating mice demonstrated that high-fat diets in reduce lipogenesis in mammary glands [45]. Opstvedt *et al*. reported that a high concentrate diet inhibited FASN activation in mammary gland [46], which is not consistent with our result, where a high concentrate diet increased, although not significantly, FASN expression in goat mammary glands, but consistent with Dong et al.’s founds that high concentrate diet increased FASN expression [22]. Further study on this issue in a more representative goat sample is required.

ACC is an enzyme that mediates the incorporation of acetate carbon into fatty acids [47]. There are two ACC isomers, ACCα (ACACA) and ACCβ, and the former is the main isomer in the liver, adipose tissue and mammary gland. Fatty acids from C4-14 are synthesized *de novo* in the mammary gland, whereas C16 arises from diet and de novo synthesis, which is performed by ACACA and FASN utilizing acetyl-CoA and butyryl-CoA [48]. Other long-chain fatty acids are predominantly derived from the diet, depending on the amount of fat in the diet [44]. Inhibition of ACACA activity does not decrease the rate of *de novo* lipogenesis, but it does attenuate the synthesis of all long chain fatty acids [49]. This is in agreement with the present study, where a high concentrate diet decreased the expression of ACACA and the concentration of long chain fatty acids in milk, such as C22:0, C20:3n3, C20:5n3 and C22:6n3. Therefore, a high concentrate diet decreased elongation by depressing the activity of ACACA.

Sterol regulatory element binding proteins (SREBPs) are transcription factors that regulate the activation of genes involved in lipogenesis and fatty acid synthesis [50]. SREBP1c is one member of this family, and it regulates many genes involved in lipid synthesis and deposition, such as FASN, LPL and SCD [51], influencing fatty acids synthesis in white adipose tissue, liver, skeletal muscle and other tissues [52]. Low availability of dietary fatty acids activates SREBP1c expression and processing, and a high fat diet decreases the expression of many genes, including LPL, FASN and SCD. Furthermore, SREBP1c-null mice fed a low-fat diet have nearly a 30% reduction in milk lipid concentration [53]. SREBP1c also plays a vital role in the adaptation of mammary lipid synthesis to low-fat diet conditions. Our high concentrate diet contained more fat, reduced the abundance of SREBP1c, although not significantly, but resulted in the low expression of genes involved in fatty acid synthesis, as demonstrated for ACACA and LPL.

In conclusion, goats fed on a high concentrate diet would suffer from SARA, which could finally affect the products of goats. A high concentrate diet had no effects on SFA, MUFA and PUFA, but reduced gene expression involved in lipids metabolism, such as ACACA, LPL, and SCD, and consequently some FA, in particular *cis*-9, *trans*-11CLA content in the milk of lactating goats, and SCD expression, which is involved in the catalysis of VA to *cis*-9, *trans*-11CLA, was significantly down-regulated. The DNA methylation rate of SCD was increased and the expression of SCD mRNA and protein was down-regulated in the mammary glands of lactating goats fed a high concentrate diet, which also reduced *cis-9*, *trans-11* CLA content in milk.
